# Layer-by-Layer-Stabilized Plasmonic Gold-Silver Nanoparticles on TiO_2_: Towards Stable Solar Active Photocatalysts

**DOI:** 10.3390/nano11102624

**Published:** 2021-10-06

**Authors:** Fons Dingenen, Natan Blommaerts, Myrthe Van Hal, Rituraj Borah, Daniel Arenas-Esteban, Silvia Lenaerts, Sara Bals, Sammy W. Verbruggen

**Affiliations:** 1 Sustainable Energy, Air & Water Technology (DuEL), University of Antwerp, Groenenborgerlaan 171, 2020 Antwerp, Belgium; fons.dingenen@uantwerpen.be (F.D.); natan.blommaerts@uantwerpen.be (N.B.); myrthe.vanhal@uantwerpen.be (M.V.H.); rituraj.borah@uantwerpen.be (R.B.); silvia.lenaerts@uantwerpen.be (S.L.); 2 NANOlab Center of Excellence, University of Antwerp, Groenenborgerlaan 171, 2020 Antwerp, Belgium; daniel.arenasesteban@uantwerpen.be (D.A.-E.); sara.bals@uantwerpen.be (S.B.); 3 Electron Microscopy for Materials Science (EMAT), University of Antwerp, Groenenborgerlaan 171, 2020 Antwerp, Belgium

**Keywords:** TiO_2_, photocatalysis, surface plasmon resonance, layer-by-layer stabilization, core-shell, solar

## Abstract

To broaden the activity window of TiO_2_, a broadband plasmonic photocatalyst has been designed and optimized. This plasmonic ‘rainbow’ photocatalyst consists of TiO_2_ modified with gold–silver composite nanoparticles of various sizes and compositions, thus inducing a broadband interaction with polychromatic solar light. However, these nanoparticles are inherently unstable, especially due to the use of silver. Hence, in this study the application of the layer-by-layer technique is introduced to create a protective polymer shell around the metal cores with a very high degree of control. Various TiO_2_ species (pure anatase, PC500, and P25) were loaded with different plasmonic metal loadings (0–2 wt %) in order to identify the most solar active composite materials. The prepared plasmonic photocatalysts were tested towards stearic acid degradation under simulated sunlight. From all materials tested, P25 + 2 wt % of plasmonic ‘rainbow’ nanoparticles proved to be the most promising (56% more efficient compared to pristine P25) and was also identified as the most cost-effective. Further, 2 wt % of layer-by-layer-stabilized ‘rainbow’ nanoparticles were loaded on P25. These layer-by-layer-stabilized metals showed superior stability under a heated oxidative atmosphere, as well as in a salt solution. Finally, the activity of the composite was almost completely retained after 1 month of aging, while the nonstabilized equivalent lost 34% of its initial activity. This work shows for the first time the synergetic application of a plasmonic ‘rainbow’ concept and the layer-by-layer stabilization technique, resulting in a promising solar active, and long-term stable photocatalyst.

## 1. Introduction

Already in the first photocatalysis studies, Fujishima and Honda (1972) pointed out the potential of TiO_2_ [1]. Its ability to produce reactive charge carriers (both conduction band electrons (e^−^_CB_) and valence band holes (h^+^_VB_)) under appropriate illumination enabled its use in several application fields, e.g., water splitting [2,3,4,5] and environmental remediation [6]. Additional advantages of TiO_2_ include its chemical stability, low cost and suitable band edge positions [7]. In contrast, the large band gap (ca. 3.2 eV) remains a major drawback. This limits the activity window to ultraviolet (UV) light which accounts for less than 5% of the incident solar spectrum on Earth [8]. Numerous strategies have been investigated to overcome this bottleneck with doping [9], sensitization [10], and heterojunctions with small band gap semiconductors [11,12]. More recently, modification of the photocatalyst surface with plasmonic metal nanoparticles has emerged as an alternative promising solution [13,14]. The deposition of plasmonic nanoparticles (NPs) like Ag and Au on TiO_2_ allows the absorption and utilization of visible (vis) light due to a unique optical property: surface plasmon resonance (SPR). SPR can be described as the collective oscillation of conduction band electrons in a metal particle. This oscillation occurs when the particle is illuminated with light of a specific wavelength [15]. The wavelength is determined by the type of metal, the size, the shape, and the dielectric environment [8]. Furthermore, the e^−^_CB_ oscillation can be energetically coupled to a semiconductor through different mechanisms [13]. Linic (2011) distinguishes three mutually nonexclusive mechanisms: (i) direct electron injection (DEI), (ii) near field enhancement (NFE) and (iii) improved scattering [16]. The latter is mainly significant for NPs larger than 50 nm [8,17]. For DEI, the excited metal electrons are transferred to the conduction band of the semiconductor. The requirement for this is a good electrochemical contact between metal and semiconductor with appropriate band alignment [18,19]. For NFE, this direct contact is not strictly required, since it enhances the activity by increasing the electrical field in the close surroundings of the plasmonic NPs [20]. Near the surface of isolated NPs, enhancements with a factor of 10^3^ are observed. These can even increase to 10^6^ if so-called ‘hot spots’ are formed, e.g., when the NPs are positioned closely (ca. 1 nm) apart. Note that the rate of charge carrier generation is proportional to the square of the electrical field [16]. An important condition for this effect is a good overlap of the absorption edge of the semiconductor and the absorption band of the plasmonic particles [7,20].

In order to respond well to both DEI and NFE, the concept of plasmonic ‘rainbow’ photocatalysis was exploited in some of our earlier work [13]. For this, bimetallic Au_x_Ag_1__−x_ NPs (x ranging from 0.2 to 1) were deposited on TiO_2_. Pure spherical Ag NPs reach an absorption maximum around 390 nm, allowing silver-rich particles to show a strong NFE effect for TiO_2_ photocatalysts [21]. On the other hand, the absorption maximum of pure spherical Au NPs lies around 520–530 nm [22]. The application of composites of both metals therefore creates a broad absorption band to generate a strong DEI effect under solar irradiation. Verbruggen et al. (2016) already showed that TiO_2_ loaded with 1.5 wt % of these bimetallic NPs outperformed pristine TiO_2_ under both simulated as well as real sunlight, under ambient conditions [13]. However, the plasmonic metal loading was not optimized, and long-term stability effects have not yet been addressed.

In particular, the stability of these metal NPs on the long term remains problematic. Especially silver-enriched particles are prone to oxidation and aggregation [23]. In that regard, several stabilization techniques have already been investigated. Two general strategies are the embedment in the photocatalyst layer [5] and the formation of protective shells (with, e.g., thiols [24], xanthate [25], and silica [20]). However, these methods do not offer full control over the thickness of the protective layer, which is of paramount importance, since the layer thickness may have a negative effect on the NFE [19]. Moreover, controlling the layer thickness on a (sub-)nano level would also allow NFE hot spot engineering. For that reason, Asapu et al. (2017) suggested the use of the layer-by-layer (LbL) method for the protection of Ag NPs [23]. They achieved ultrastability for pure spherical Ag NPs by applying alternating layers of positively and negatively charged polyelectrolytes, polyallylamine hydrochloride (PAH) and polyacrylic acid (PAA), respectively. Since the adhesion of the layers is mostly based on coulombic interaction, the thickness of the protective shell around the plasmonic metal core can be controlled at a sub-nanometer level.

In this study the plasmonic ‘rainbow’ concept for broadband solar photocatalysis, and the LbL technique for long-term stability, are merged for the first time. First of all, the plasmonic ‘rainbow’ photocatalyst was optimized towards the type of TiO_2_ support, as well as the metal loading. The most photoactive composite under solar light was identified by evaluating the photocatalytic performance of various ‘rainbow’–TiO_2_ composites under air mass (AM) 1.5G simulated sunlight. A brief techno-economic analysis was conducted, showing the cost-effectiveness of the modified species. Secondly, the bimetallic Au_x_Ag_1__−x_ NPs were stabilized using the LbL technique. The effect of this protective shell was studied in two harsh environments (saline water and hot air), and the result on the long-term activity was evaluated. Furthermore, computational electromagnetic simulations were used to gain insight in the electrical near field change after applying the stabilizing shells.

## 2. Materials and Methods

### 2.1. Synthesis and Characterization of Bare Plasmonic ‘Rainbow’ Photocatalysts

Nine colloidal spherical Au_x_Ag_1__−x_ NPs suspensions (x ranging from 0.2 to 1, incremental steps of 0.1) were synthesized using the modified Turkevich method described elsewhere [13,26]. The ‘rainbow’ mixture was produced by taking equal amounts of each suspension.

The Au_x_Ag_1__−x_ NPs were characterized with UV–vis spectroscopy and a Spectroquant^®^ elemental analysis. The former allowed to investigate the plasmon absorption bands in water using a Shimadzu UV–vis 2501 PC double beam spectrophotometer in a 300–800 nm range at a resolution of 0.2 nm. The latter served to accurately quantify the gold (NOVA 60, Merck (Darmstadt, Germany), 114 821 test kit) and silver (NOVA 60, Merck, 114 831) content of the samples.

Three different TiO_2_ substrates were loaded with different plasmonic ‘rainbow’ (R) loadings (0 wt %, 1 wt %, and 2 wt % R). The studied TiO_2_ species were pure anatase (Sigma-Aldrich, Steinheim, Germany, >99.8%), PC500 (CristalActive, ~85% anatase, ~15% amorphous) and P25 (ACROS Organics, Geel, Belgium, ≥99.5%, ~80% anatase, ~20% rutile). The loading was established by photoimpregnation under UV-A illumination (Philips Cleo UVA, 25 W, 365 nm) and vigorous stirring during at least 1.5 h. The resulting suspension was centrifuged (10,000× *g*; 15 min), washed and dried overnight at 105 °C. The dried powder was crushed using pestle and mortar.

The plasmonic enhanced TiO_2_ samples were further characterized using N_2_ sorption and diffuse reflectance spectroscopy (DRS). N_2_ sorption was applied to perform a Brunauer–Emmett–Teller (BET) analysis using Micromeritics’ TriStar 3000 V6.04 A after degassing overnight at 200 °C. DRS spectra were recorded with the Shimadzu UV–vis 2501 PC double beam spectrophotometer, equipped with a 60 mm BaSO_4_-coated integrated sphere and a photomultiplier R-446 detector in reflection mode. BaSO_4_ was used here as carrier material and blanc. From the DRS spectra, the band gaps were calculated using a Kubelka–Munk transformation.

### 2.2. Synthesis and Characterization of LbL-Stabilized Plasmonic Photocatalysts

For efficiency reasons, two compositions, Au_0.3_Ag_0.7_ and Au_0.7_Ag_0.3_, were stabilized using the LbL method. These were selected since their 1:1 combined absorbance spectrum matches the solar spectrum on Earth well. In both cases, 4 protective layers (2 x PAH (Sigma-Aldrich, Steinheim, Germany; 17.5 kDa) and 2 x PAA (Sigma-Aldrich, Steinheim, Germany; 2 kDa)) were used by analogy with the work of Asapu et al. (2017) [23]. This protocol was adjusted for Au_0.3_Ag_0.7_ and Au_0.7_Ag_0.3_ by changing the centrifugation settings to 4500× *g* and 3500× *g* for 30 min, respectively. The loading of these NPs was again established by performing a photoimpregnation like in Section 2.1. A UV–vis absorption spectrum (Shimadzu UV–vis 2501 PC double beam spectrophotometer) was recorded after the deposition of each new layer to follow the redshifts. The final concentrations were determined using a Spectroquant^®^ elemental analysis. The shell thickness was evaluated with bright field transmission electron microscopy (BF-TEM) using an aberration-corrected cubed FEI Titan microscope operating at 300 kV. Finally, the loading of the LbL-stabilized NPs on TiO_2_ was again established by performing a photoimpregnation like in Section 2.1. The ultimate metal loading was verified by high-angle annular dark field scanning transmission electron microscopy (HAADF-STEM) and energy dispersive X-ray spectroscopy (EDS) using an aberration-corrected cubed FEI Titan microscope equipped with a Super X EDS detector operating at 300 kV. The operational conditions for all electron microscopy studies are described elsewhere [27].

### 2.3. Stabilization Testing

The success of the stabilization procedure was evaluated by a salt addition and hot air experiment. For the former, 150 µL of a 1 M NaCl solution was added to 1.5 mL of both original and LbL-stabilized plasmonic NP suspensions. All concentrations were equalized by using the Spectroquant^®^ data. UV-vis absorbance was quantified before and after the salt addition with a Shimadzu UV–vis 2501 PC double beam spectrophotometer. The oxidation of the particles was also forced by a hot air treatment. Concentrated original and LbL-stabilized plasmonic NPs were therefore drop casted on a glass substrate prior spin coated with a 1 wt % PC500 in ethanol solution (1000 rpm, 1 min, Laurell Technology Corporation spin coater) and left in the presence of air at 100 °C for 24 h.

### 2.4. Photocatalytic Activity Testing

The general protocol for the stearic acid (SA) degradation experiment is described in our previous works [13,17] and is based on the early work of Paz et al. (1995) [28]. In brief, the samples were prepared by drop casting 50 µL of a 0.5 wt % photocatalyst suspension in ethanol on a precleaned silicon wafer (1.5 cm × 3 cm). This procedure led to a photocatalyst loading of 44 µg cm^−2^. Both LbL-stabilized and bare plasmonic ‘rainbow’ photocatalysts were investigated. Empty reference samples and wafers containing only plasmonic NPs without TiO_2_ were taken along in the analysis as well as control samples. These served to study the effect of direct photolysis and direct plasmonic effects (heating, oxidation). After drying overnight at 105 °C, 100 µL of a 0.25 wt % SA (Sigma–Aldrich, ≥99.5%) in chloroform solution were spin coated on the wafers. A Laurell Technology Corporation spin coater was used for this at a speed of 1000 rpm during 1 min. Finally, the samples were dried for 20 min at 105 °C after which they were allowed to acclimatize at room temperature in the dark.

The SA was degraded by the photocatalysts under 100 mW·cm^−2^ simulated sunlight (Figure S1) The used light for this was a 300 W Xe lamp (Oriel Instruments) equipped with an AM 1.5G (Air Mass 1.5 global spectrum) filter. Its intensity at the sample position was verified by a calibrated spectroradiometer (Avantes Avaspec-3648-USB2). The degradation itself was monitored by Fourier transform infrared (FTIR) spectroscopy using a Nicolet^TM^ 380 (Thermo Fisher Scientific) with ZnSe windows at a resolution of 1 cm^−1^. The samples were positioned at a vertical angle of 9° in order to minimize internal reflections. The SA concentration was determined by integration over the wavelength range 2800–3000 cm^−1^, corresponding to a symmetric ν_s_(CH_2_) in-plane C-H stretch at 2853 cm^−1^, an asymmetric ν_as_(CH_2_) in-plane C-H stretch at 2923 cm^−1^ and the asymmetric ν_as_(CH_3_) in-plane C-H stretch at 2958 cm^−1^ [29,30]. One unit of integrated absorbance corresponded here to 1.39 × 10^16^ SA molecules per cm^2^, as determined by a calibration curve from earlier work (*R*^2^ = 0.99) [17]. Both the northern and southern parts of the wafer were tested. The measurements were stopped if an integrated absorbance of ~0.3, corresponding to 4.10^15^ SA molecules·cm^−2^, was attained.

To study the stability, aging tests were executed as well. For this, both LbL-stabilized as their non-LbL equivalent samples were re-tested after 1 month of aging open to the air. Numerical simulations based on Maxwell’s equations using COMSOL Multiphysics were performed to provide a complementary insight into the effect of the shell on the resulting field enhancement. Detailed descriptions of the finite element models (FEMs) are given in Appendix A. Finally, to determine the intrinsic photostability of the polymer shell itself, the LbL-stabilized samples were illuminated in a slit-shaped flatbed reactor closed system while monitoring the CO_2_ formation using FTIR spectroscopy [31,32].

## 3. Results

### 3.1. Pristine Plasmonic ‘Rainbow’ Photocatalysts

**Figure 1:**
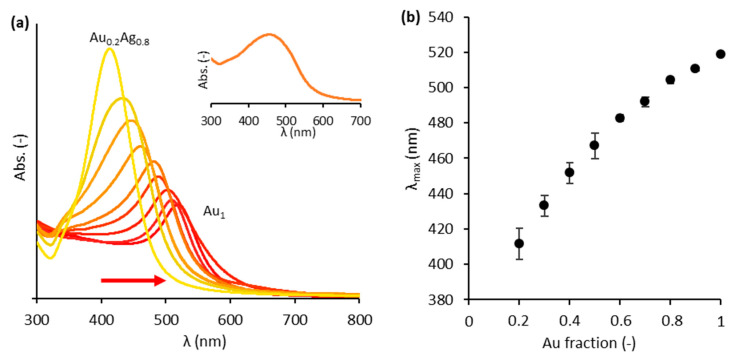
(**a**) UV–vis absorbance spectra for each composition of Au_x_Ag_1__−x_ suspensions (x ranging from 0.2 to 1 with incremental steps of 0.1). The colors of the curves correspond to the colors of the actual colloids. The arrow indicates the redshift as the Au content increases. Inset: UV–vis absorbance spectrum for the total (‘rainbow’) mixture. (**b**) The wavelengths of maximal absorbance for the different suspensions. The error bars represent the standard deviation for the 4 times the suspensions were synthesized.

The colloidal Au_x_Ag_1__−x_ NPs (Figure S2) were successfully synthesized using the modified Turkevich method [13,26]. Both UV-vis spectroscopy and a Spectroquant^®^ elemental analysis were used to characterize the NPs. First, the absorbance of each composition is depicted in Figure 1a, clearly indicating a redshift as the Au content increases. However, the wavelength of maximal absorbance (λ_max_) does not vary perfectly linearly with the Au content (Figure 1b). It is noticed that λ_max_ decreases more rapidly when moving towards lower Au contents. This can be explained by the fact that these bimetallic particles are not perfect alloys. Blommaerts et al. (2019) showed that these Au_x_Ag_1__−x_ NPs consist of a gold-enriched core and a silver-enriched shell, due to the formation mechanism of the modified Turkevich synthesis based on gold nucleus seeds [33]. Borah et al. (2020) recently revealed a similar progressive decrease in λ_max_ at lower Au contents for 60 nm Au@Ag core-shell NPs using numerical simulations [34]. The linear combination of all NPs provides a valuable strategy to achieve a strong optic response and a broad absorption band as shown in the inset of Figure 1a. Secondly, the Spectroquant^®^ analysis revealed that the theoretical and experimental gold and silver content in the suspensions never differed by more than 2% (Table S1).

Plasmonic ‘rainbow’ photocatalysts were subsequently synthesized using pure anatase, PC500, and P25 as supports, with a ‘rainbow’ (R) NP loading of 0, 1, and 2 wt % R. While the synthesis was always successful for anatase and P25, PC500 could not be photoimpregnated successfully. This is also confirmed by DRS spectra (Figure S3) which show only a very poor vis absorption for modified PC500. We assume that the generated charge carriers in PC500 during the UV-A illumination step of the photoimpregnation procedure are too unstable to effectively expel the solvent and citrate molecules required to attach the metal NPs.

The DRS spectra were used to calculate the band gap (Table 1) after a Kubelka–Munk transformation. Importantly, although plasmonic NPs assure absorption in the vis region, they leave the band gap of the semiconducting substrate unaffected, still corresponding to the UV region (3.1–3.4 eV). Table 1 displays the results of the N_2_ sorption/desorption analysis as well. N_2_ sorption curves are given in Figure S4. From this, it clearly appears that PC500 has the largest specific surface area (ca. 293 m^2^ g^−1^) compared to anatase (ca. 68 m^2^ g^−1^) and P25 (ca. 50 m^2^ g^−1^) which is consistent with the literature [35].

### 3.2. LbL Stabilization of Bimetallic NPs

**Figure 2:**
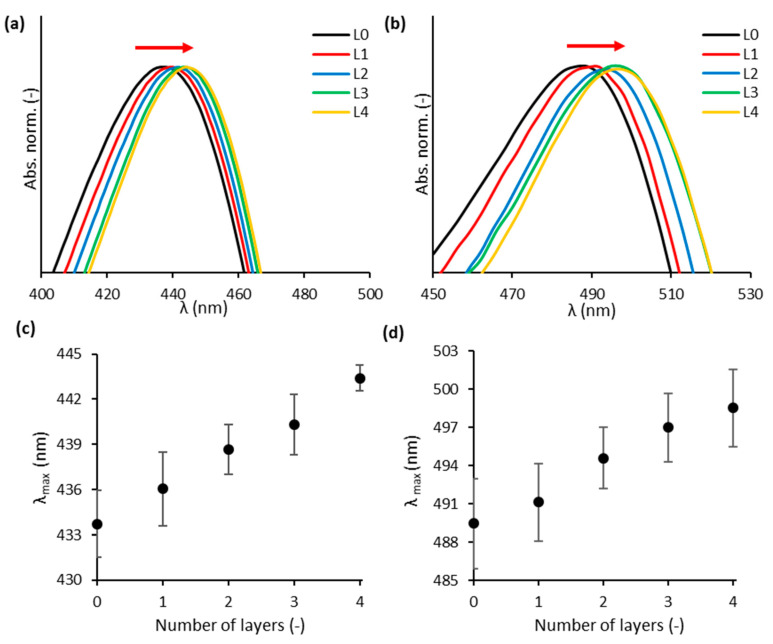
Details of normalized UV–vis absorbance (Abs. norm.) spectra for the (**a**) Au_0.3_Ag_0.7_ and (**b**) Au_0.7_Ag_0.3_ NP suspensions for different numbers of applied layers (L). The wavelength of maximal absorbance (λ_max_) for the (**c**) Au_0.3_Ag_0.7_ and (**d**) Au_0.7_Ag_0.3_ NP suspensions with different numbers of layers. The error bars, based on the standard deviation, predominantly reflect the variability in λ_max_ for bare NPs, and not the variability in redshift with increasing layer number.

Au_0.3_Ag_0.7_ and Au_0.7_Ag_0.3_ were stabilized using the LbL method, based on alternate deposition of positively and negatively charged polyelectrolytes, adapted from Asapu et al. (2017) [23]. A redshift is observed in the absorption spectra (Figure 2a,b) as the number of polyelectrolyte layers increases. This can be explained by the change in the direct dielectric environment due to the new polymer layers. The redshifts are of the order of 1–3 nm (Figure 2c,d) which is slightly higher than the reported values of 1 nm for 20 nm pure gold and silver NPs [19,23]. 

**Figure 3:**
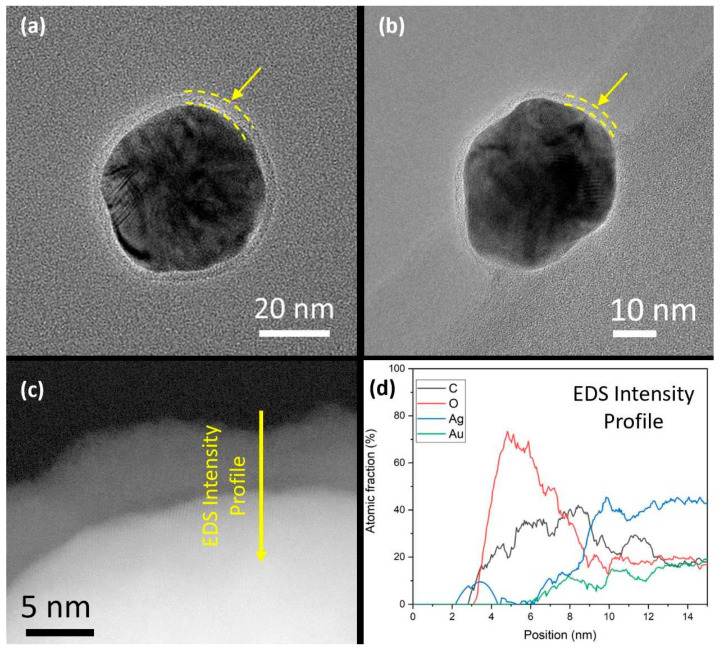
BF-TEM images of LbL-stabilized (**a**) Au_0.3_Ag_0.7_ and (**b**) Au_0.7_Ag_0.3_ NPs. The yellow arrows and dashed lines indicate the presence of a thin and homogeneous polymer shell around the metal cores. (**c**) High resolution HAADF-STEM image from a detail of a nanoparticle from the Au_0.3_Ag_0.7_ sample where the LbL shell is clearly observed after 10 months and (**d**) EDS line intensity profile which indicates the polymeric composition of the shell.

Using BF-TEM, the final shell thickness after the deposition of four layers was determined to be 4 ± 1 nm and 2.3 ± 0.5 nm for Au_0.3_Ag_0.7_ and Au_0.7_Ag_0.3_ NPs, respectively (Figure 3a,b). Further analysis performed on the Au_0.3_Ag_0.7_ nanoparticles after 10 months shows a great stability of the nanoparticles. EDS analysis confirms that the polymeric coating remains stable and continues protecting the bimetallic nanoparticles from degradation (Figure 3c,d). Note that the bare Au_0.3_Ag_0.7_ NPs are larger in size compared to the Au_0.7_Ag_0.3_ NPs (49 ± 4 nm vs. 32 ± 5 nm), which is consistent with the literature [17].

**Figure 4:**
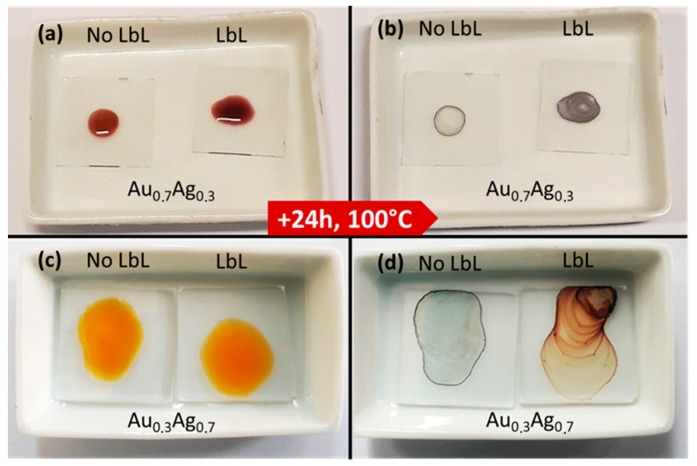
Concentrated droplets on a glass substrate of LbL-stabilized and nonstabilized Au_0.7_A_0.3_ (**a**,**b**) and Au_0.3_Ag_0.7_ (**c**,**d**), before (**a**,**c**) and after 24 h (**b**,**d**), at 100 °C.

**Figure 5:**
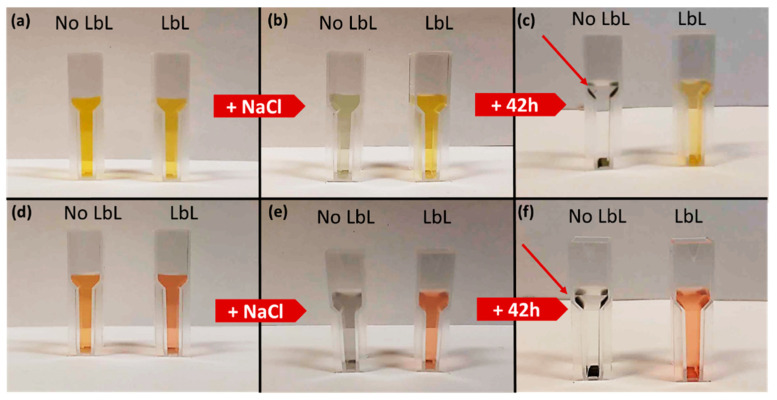
A total of 1.5 mL of LbL and non-LbL-stabilized Au_0.3_Ag_0.7_ and Au_0.7_Ag_0.3_ suspensions before (**a**,**d**), immediately after (**b**,**e**) and 42 h after (**c**,**f**) the addition of 150 µL 1 M NaCl solution. The red arrows indicate the black precipitates formed by oxidation and aggregation of the deactivated metal NPs.

**Figure 6:**
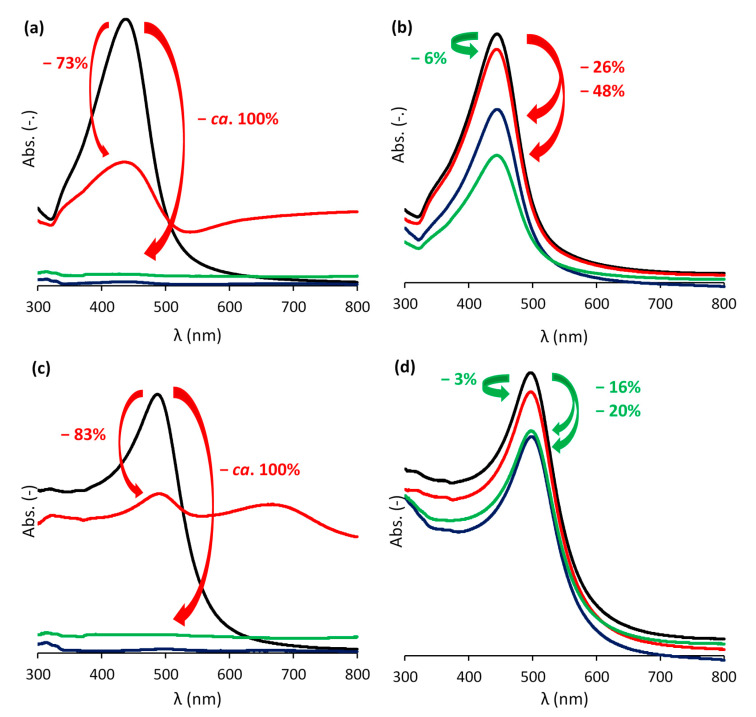
UV-vis absorbance spectra for both non-stabilized (**a**,**c**) and LbL-stabilized (**b**,**d**) Au_0.3_Ag_0.7_ (**a**,**b**) and Au_0.7_Ag_0.3_ (**c**,**d**) NP suspensions before (black), immediately (red), 24 h (blue), and 42 h (green) after the addition of 150 µL of a 1 M NaCl solution to 1.5 mL NP suspension. The values and arrows in green and red indicate the decrease of the initial maximal absorbance, corrected for dilution and formation of degradation products.

The stability of the LbL-stabilized plasmonic NPs was demonstrated by a hot air treatment and a salt addition test. The hot air treatment showed the superior stability of the LbL-stabilized particles, since their color was retained even after prolonged exposure to oxygen at elevated temperatures (Figure 4a–d). Figure 5a–f depicts the suspensions before and after adding NaCl in the salt addition test. While the suspensions of LbL-stabilized NPs retain their color, an immediate discoloration was observed in the absence of the stabilizing LbL shells. This discoloration originates from the aggregation (and oxidation) of the particles due to the neutralization of the negatively charged citrate ligands surrounding the NPs after the Turkevich synthesis. As a consequence, the electrostatic repulsion between the particles disappears and the particles aggregate. Only after 42 h, the color of the LbL-Au_0.3_Ag_0.7_ suspension also vanished just slightly (Figure 5c). The extent of discoloration was quantified by measuring the maximal absorbance before and after the NaCl addition and correcting for dilution effects (Figure 6a–d). For Au_0.3_Ag_0.7_ NPs, the LbL-stabilized NPs lost barely 6% of their maximal absorbance, while in the case of bare NPs approximately 73% of the absorbance was lost and extra bands appeared in the absorption spectrum. After 24 h and 42 h, the maximum absorption of bare NP samples decreased to 98% and 99%, respectively. Much smaller absorbance decreases were recorded for the LbL-Au_0.3_Ag_0.7_ suspension, amounting to 26% and 48% after 24 h and 42 h, respectively. The LbL-stabilized Au_0.7_Ag_0.3_ NPs perform significantly better: 97% was retained immediately after addition, lowering to 84% and 80% after 24 h and 42 h, respectively. The original bare Au_0.7_Ag_0.3_ suspension without stabilization only retained ca. 17% of its maximal absorbance immediately after salt addition, further decreasing to 4% (24 h) and an insignificant 2% after 42 h. In order to increase the stability, more layers could be applied, but at the cost of a lowered near field enhancement (vide infra).

### 3.3. Photocatalytic Activity

#### 3.3.1. Pristine Plasmonic ‘Rainbow’ Photocatalysts

**Figure 7:**
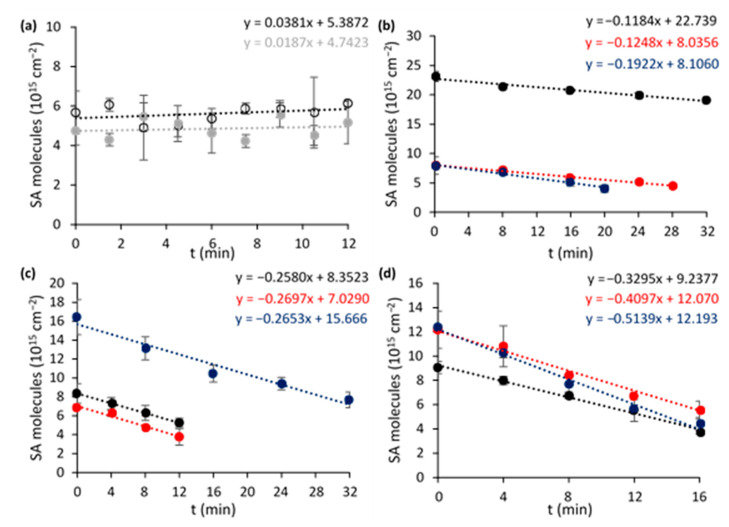
SA degradation curves for (**a**) an empty Si wafer (hollow black) and a Si wafer with only plasmonic rainbow NPs (0.88 µg cm^−2^, grey), (**b**) pure anatase, (**c**) PC500 and (**d**) P25 samples with 0 wt % R (black), 1 wt % R (red), and 2 wt % R (blue). Error bars are based on the mean deviations from the north and south side of the sample.

**Figure 8:**
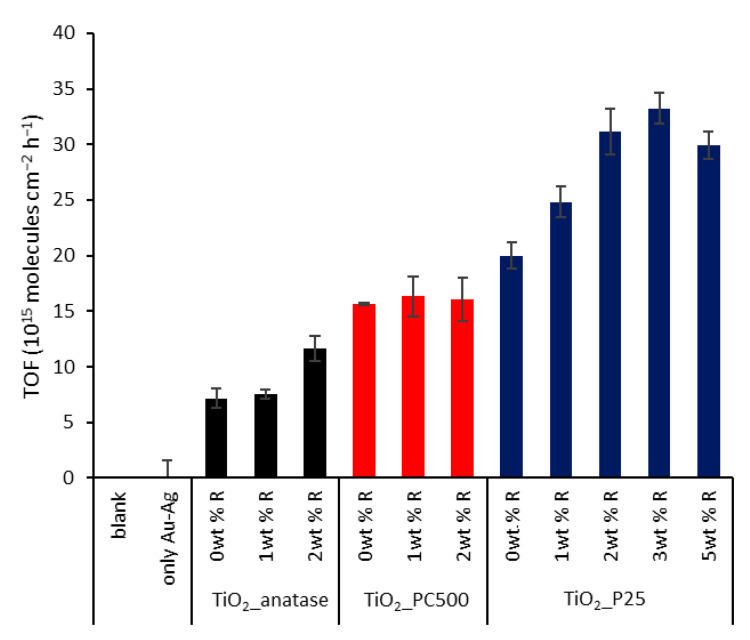
Summary of the turnover frequencies (TOFs) for all samples. The error bars are based on the standard errors of the SA degradation curve slopes.

First, the most optimal ‘rainbow’ NP loading and TiO_2_ support were determined by comparing different composites towards photocatalytic SA degradation under AM1.5G simulated sunlight. This reaction shows zero-order reaction kinetics for flat nonporous films [36] and therefore does not depend on the initial SA concentration. The degradation curves obtained for the different modified TiO_2_ species are presented in Figure 7a–d and a summary of the corresponding turnover frequencies (TOFs) is shown in Figure 8. The control experiments using a blank wafer and a sample containing only Au_x_Ag_1__−x_ NPs (0.88 µg cm^−2^) (Figure 7a) show that direct photolysis, direct plasmonic heating or oxidation do not contribute significantly under the present conditions. Furthermore, it is shown that pristine P25 outperforms unmodified pure anatase (+160%) and PC500 (+31%). This may be explained by P25’s heterostructure of rutile and anatase, resulting in a more efficient charge separation. This enables more efficient use of the UV light component that is present in sunlight [37]. Su et al. (2011) demonstrated further that the synergetic effect is mostly present for composites with a rutile content of ≥20% and an anatase fraction of ≥40%, explaining P25’s superiority [38]. In addition, the presence of localized electronic states at the interface between rutile and anatase also induces weak activity under visible light [37]. The reason why unmodified PC500 performs better than anatase is because of its larger specific surface area (296 m^2^ g^−1^ vs. 69 m^2^ g^−1^).

The plasmonic enhancement is clear for pure anatase and P25. As expected, the unsuccessful plasmon loading for PC500 leads to similar degradation rates for all PC500 species. The best results are recorded for plasmon-enhanced P25, in particular P25 + 2 wt % R (56% increase compared to pristine P25). To identify the optimal loading, also P25 + 3 wt % R and + 5 wt % R were synthesized, characterized, and tested. They showed similar band gaps (3.22 and 3.19 eV, respectively) and specific surface areas (48.8 ± 0.4 and 48.5 ± 0.2 m^2^ g^−1^) as the other P25 species (Figure S5). However, it was observed that the photocatalytic activity towards SA degradation leveled off and even decreased for the case of 5 wt % R, with +66% and +50%, respectively, compared to pristine P25 (Figure S6). This can be explained by a higher occupation of active sites on the titania, but more likely the higher absorption and scattering of UV light by the plasmonic particles plays a major role. The interband absorption of UV light by the metals can become especially problematic at excessive loadings.

The optimal loading was further determined by means of a simplified technoeconomic analysis using the incremental cost-effectiveness ratio (ICER) (Equation (1)) [39].

(1) $$\mathrm{ICER}=\frac{∆{\mathrm{Cost}}_{\mathrm{metal}\;\mathrm{loading}}}{∆\mathrm{TOF}\;}$$

**Figure 9:**
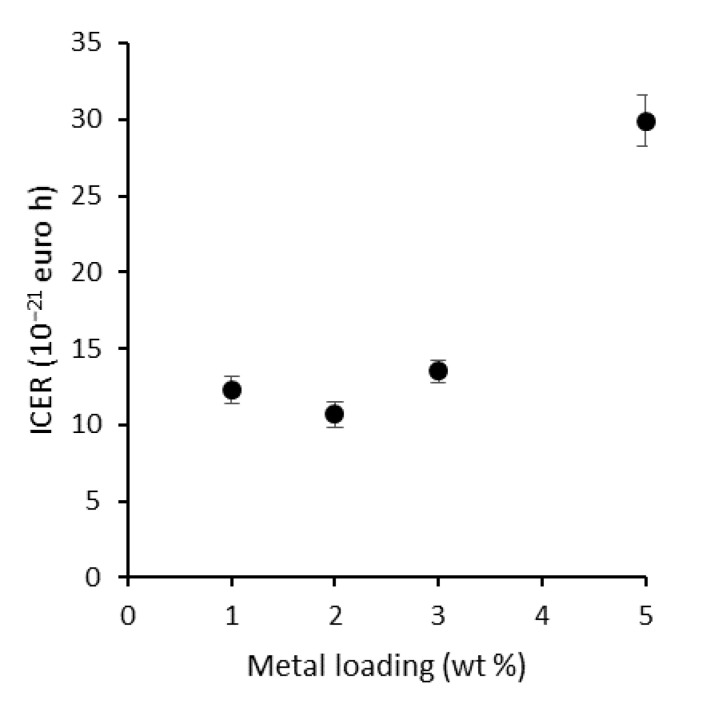
ICER plotted against the metal loading of P25, showing that the economic optimum for plasmon modification is situated at 2 wt % R.

With ∆Cost_metal loading_ the difference in cost of the used plasmonic NPs based on the cost of gold and silver precursors at the time of preparation (EUR 91.6 g^−1^ [40] and EUR 2.68 g^−1^ [41], respectively). ∆TOF is the difference in the corresponding TOFs. The lowest ICER value is thus preferable, as it corresponds to a small increase in cost for a large gain in TOF. In that regard, P25 + 2 wt % R proved to be the optimal combination (Table S2 and Figure 9). Consequently, this loading is used for the fabrication of a LbL-stabilized ‘rainbow’ sample. An important remark is that further economical optimization can be done by using more silver-rich particles due to the significantly higher cost of gold (precursor). In addition, more silver-rich particles could benefit more from the NFE mechanism due to the larger overlap with the absorption edge of TiO_2_, potentially leading to even higher activities. This provides an important perspective for further research, if their long-term stability is addressed appropriately.

#### 3.3.2. LbL-Stabilized Plasmonic Photocatalysts

**Figure 10:**
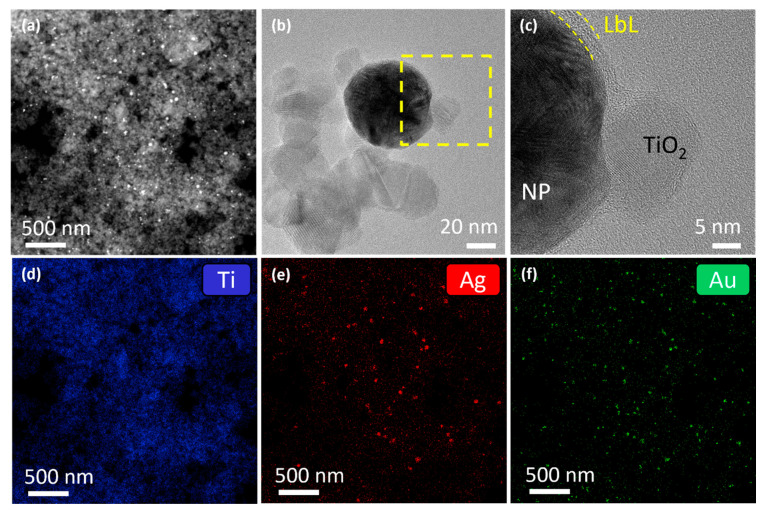
(**a**) HAADF-STEM image of the P25 material loaded with 2 wt % LbL-stabilized Au_0.3_Ag_0.7_ + Au_0.7_Ag_0.3_ NPs. High resolution TEM image from (**b**) a detail of the union of an alloy particle with the TiO_2_ nanoparticles and (**c**) a magnified area indicated in the yellow square in (**b**) where it can be observed that the particles keep their polymeric shell. EDS maps of the area showed on (**a**) for (**d**) Ti, (**e**) Ag, and (**f**) Au.

To study the effect of LbL stabilization on the photocatalytic activity of gold-silver rainbow catalysts, P25 was loaded with 2 wt % LbL-stabilized Au_0.3_Ag_0.7_ + Au_0.7_Ag_0.3_ NPs, as well as 2 wt % of pristine NPs. Characterization by DRS spectroscopy (Figure S7) and BET analysis (Figure S8), showed similar results to those of the other plasmonic modified P25 samples. The HAADF-STEM image of the LbL-stabilized sample showed a uniform distribution of NPs over the P25 surface (Figure 10a). High resolution TEM images of a magnified area demonstrate that the NPs retain their polymeric shell, which additionally seems to facilitate anchoring to the TiO_2_ nanoparticles (Figure 10b,c). EDS maps display a homogeneous distribution of both LbL-stabilized Au_0.3_Ag_0.7_ and Au_0.7_Ag_0.3_ NPs (Figure 10d–f). The experimentally determined total metal loading by EDS reveals just a slightly lower value than the nominal loading (1.6 ± 0.2 wt % vs. 2 wt %) with Ag and Au approximately present in equimolar amounts (0.14 ± 0.03 mol% Ag and 0.17 ± 0.02 mol% Au, Table S3).

**Figure 11:**
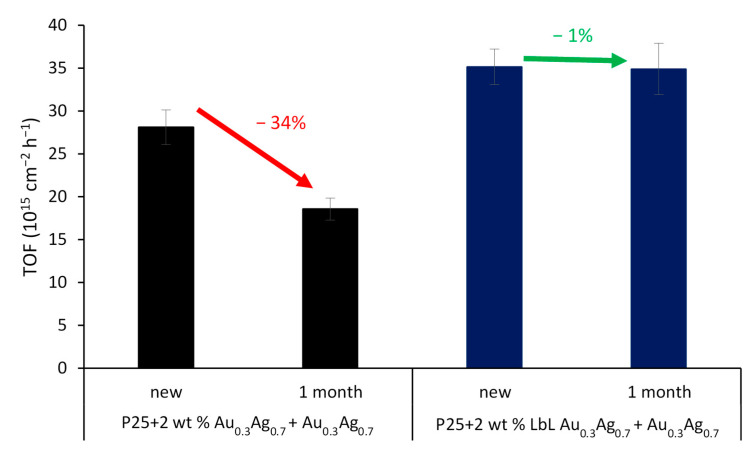
TOF for SA degradation for P25 + 2 wt % Au_0.3_Ag_0.7_ + Au_0.7_Ag_0.3_ (black) and its LbL-stabilized equivalent (blue), both tested new and after 1 month in darkness. The error bars are based on the mean deviations of the different samples.

**Figure 12:**
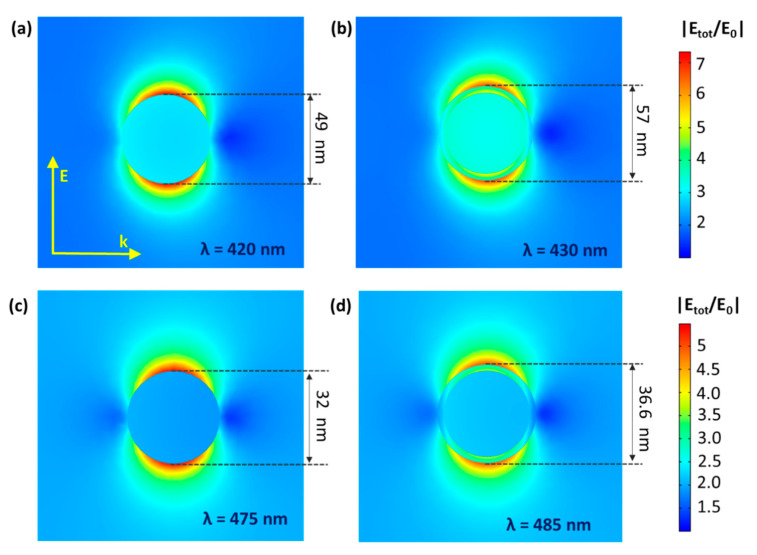
NFE maps for original (**a**,**c**) and LbL-stabilized (**b**,**d**) Au_0.3_Ag_0.7_ (**a**,**b**) and Au_0.7_Ag_0.3_ (**c**,**d**), plotting the ratio between the total electrical near field and the incident electrical field (|E_tot_/E_0_|) for the wavelength (λ) of maximal NFE. Note that the enhancement of TiO_2_’s activity by the near field occurs proportionally to |E_tot_/E_0_|^2^. Both the direction of the wave (k) and energy vector (E) are given as well.

Interestingly, for the SA degradation, the LbL-stabilized catalyst displays a significantly higher activity than the non-LbL-stabilized equivalent (+23%) (Figure 11, SA degradation curves displayed in Figure S9). The effect of the polymer shell is far from straightforward. On the one hand, the electrically insulating polymer layer eliminates the DEI pathway; on the other hand, it also affects the electrical near field at the same time. As shown by Asapu et al. (2017), the latter effect is especially important in this reaction [19]. In general, the electrical near field around a plasmonic nanoparticle in vacuum (or any medium) decreases with increasing distance from the NP surface. This near field is intensified by the presence of an optimally thin dielectric shell around the nanoparticle due to its polarizability resulting from the bound electrons. The intensification of the near field increases with the dielectric constant (ε), thus favoring the polymers over air (ε >2 vs. ~1 for air at 25 °C in the UV–vis light frequency range) [42]. Importantly, upon increase of the dielectric shell thickness beyond the optimum, the near field amplitude again drops proportionately, since the distance effect becomes dominant. While experimental measurement of the near field enhancement is a cumbersome task, classical electromagnetic calculations with suitable dielectric properties can very conveniently estimate the near field amplitudes for different scenarios and facilitate explicit comparisons. Electromagnetic simulations were therefore performed using COMSOL Multiphysics to quantify the NFE for the isolated plasmonic NPs (Figure 12a–d). It is shown that the NFE, given as the ratio between the total and the incident electrical field (|E_tot_/E_0_|) averaged over the NP surface, only slightly changes for the shell thicknesses applied in this study. After LbL stabilization, the NFE spectra experience a small redshift, but the maximum values only vary by −2% and +1% for Au_0.3_Ag_0.7_ and Au_0.7_Ag_0.3_ NPs, respectively (Figure S10). The difference between them can be explained by the larger shells of the former. The decrease for the 4 nm shell is still limited, consistent with Yüksel et al. (2020) [43]. They determined that for 50 nm Au NPs the NFE dropped dramatically for shell thicknesses ≥5 nm. Furthermore, the activity increase for the LbL-stabilized samples may originate from a higher resistance against the rather harsh drying conditions (105 °C, overnight) during the catalyst synthesis procedure. This most likely results in the oxidation of the plasmonic NPs to a certain extent. As mentioned earlier (see Section 3.1) Blommaerts et al. (2019) already demonstrated that the outer parts of the bimetallic particles are enriched in silver, being the less stable element [33]. Several studies point out a significant NFE decrease of about one order of magnitude or higher for a thin diffuse AgO_2_ layer (≤ 2 nm) [23,44]. This oxidation causes the further deactivation of the non-LbL-stabilized samples. After 1 month of aging open to the air, these samples lost ca. 34% of their initial activity, which is lower than that of pristine P25. On the other hand, the LbL samples retained over 99% of their initial activity, which is a highly promising result. This convincingly confirms the stabilizing effect of the protective polymer shell. Furthermore, FTIR studies in a closed system show no significant degradation of the polymer shell itself upon prolonged irradiation (Figure S11). In future research, the activity of such stabilized plasmon-enhanced TiO_2_ may be further improved by using conductive polymer shells (e.g., polyaniline [19]). In this way, the DEI mechanism would not be inhibited.

## 4. Conclusions

In order to increase the photocatalytic activity of TiO_2_ under solar light, the use of the plasmonic ‘rainbow’ concept was further explored. Three different TiO_2_ supports (pure anatase, PC500, and P25) were loaded with 0, 1, and 2 wt % Au_x_Ag_1__−x_ NPs, with x ranging from 0.2 to 1. The most optimal composite towards photocatalytic stearic acid degradation under AM1.5G simulated sunlight was determined to be P25 + 2 wt % ‘rainbow’ NPs. A simplified technoeconomic calculation also confirmed this to be the most cost-effective sample. Additionally, the poor stability of gold–silver NPs was addressed by protecting the particles by an ultrathin polymer shell applied using the layer-by-layer technique. Hot air treatment and salt addition experiments demonstrated the superior stability of the NPs subjected to this stabilization procedure. Electromagnetic simulations showed a negligible change in near field enhancement for the isolated plasmonic NPs, due to the competition between an adverse distance effect and a beneficial dielectric polarization effect. Finally, an aging experiment with P25 + 2 wt % LbL-stabilized Au_x_Ag_1__−x_ confirmed the superior stability and retained activity after 1 month, with >99% retention of the initial activity. This shows the potential of modifying TiO_2_ with LbL-stabilized plasmonic Au_x_Ag_1__−x_ NPs for long-term stable solar light photocatalysis.

## Figures and Tables

**Table 1:** Band gaps (E_g_) and specific surface area (A) for the different plasmonic ‘rainbow’ catalysts.

Metal Loading (wt %)	TiO_2__Anatase		TiO_2__PC500		TiO_2__P25	
	E_g_ (eV)	A (m^2^ g^−1^)	E_g_ (eV)	A (m^2^ g^−1^)	E_g_ (eV)	A (m^2^ g^−1^)
0	3.16	72.2	3.36	296	3.27	51.8
1	3.14	66.7	3.42	291	3.17	50.8
2	3.15	68.9	3.38	302	3.28	49.4

## Data Availability

The data presented in this study are available on request from the corresponding author.

## Supplementary Materials

The following are available online at https://www.mdpi.com/article/10.3390/nano11102624/s1, Figure S1: The irradiance spectrum of the used solar simulator, Figure S2: The different Au_x_Ag_1__−__x_ NP suspensions, Figure S3: DRS spectra for (a) pure anatase species, (b) PC500 species and (c) P25 species, plotting the fraction of reflectance (R%) against the wavelength of the incident light (λ), Figure S4: N_2_ adsorption (black) and desorption (red) curve, plotting the adsorbed quantity (in cm^3^·g at standard temperature and pressure (STP)) vs. the pressure ratio (P/P_0_), for a representative (a) pure anatase, (b) PC500, and (c) P25 sample, Figure S5: DRS spectra for P25 + 2 wt % R (black), + 3 wt %. R (red) and + 5 wt % R (blue), plotting the fraction of reflectance (R%) against the wavelength of the incident light (λ). Figure S6: SA degradation curves, plotting the number of SA molecules per cm^2^ against the time (t) for P25 + 2 wt % R (black), + 3 wt % R (red) and + 5 wt % R (blue), Figure S7: DRS spectra for P25 + 2 wt % R (black) and P25 + 2 wt % non- (red) and LbL-stabilized (blue) Au_0.3_Ag_0.7_ + Au_0.7_Ag_0.3_, plotting the fraction of reflectance (R%) against the wavelength of the incident light (λ), Figure S8: N_2_ adsorption curve for both P25 + 2 wt % non- (red) and LbL-stabilized (blue) Au_0.3_Ag_0.7_ + Au_0.7_Ag_0.3_, plotting the adsorbed volume (V) at standard temperature and pressure (room temperature and 1 atm) against the relative pressure (P/P_0_, ranging between 0 and 0.3), Figure S9: SA degradation curves, plotting the number of SA molecules per cm^2^ against the time (t) for a representative P25 + 2 wt % Au_0.3_Ag_0.7_ + Au_0.7_Ag_0.3_ sample (red) and the LbL-stabilized equivalent (blue) samples, Figure S10: Near field enhancement (NFE), expressed as the ratio between the scattered electrical near field and the incoming electrical field (|E_tot_/E_inc_|), for both isolated original (full) and LbL-stabilized (dashed) Au_0.3_Ag_0.7_ (black) and Au_0.7_Ag_0.3_ (red), plotted against the wavelength of the incoming light, Figure S11: CO_2_ FTIR absorbance plotted against time in a closed flatbed reactor with P25 + 2 wt % LbL-Au_0.3_Ag_0.3_ + -Au_0.7_Ag_0.3_ (44 µg cm^−2^, 3 cm × 1.5 cm) for a 497 mL·min^−1^ air flow, Table S1: Theoretical gold fraction (Au%_th_) and experimental gold (Au%_m_) and silver fractions (Ag%_m_) for the different Au_x_Ag_1__−x_ NP suspensions, Table S2: Used metal masses, corresponding costs for a Au and Ag precursor price of EUR 91.6 g^−1^ and EUR 2.68 g^−1^, respectively, TOFs and ICERs for the different metal loadings of P25 (for 1 m^2^ with a photocatalyst loading of 44 µg cm^−2^), Table S3: Experimental (Exp.) atomic fraction and experimental and theoretical (Th.) mass fraction for O, Ti, Ag, and Au in P25 + 2 wt % LbL Au_0.3_Ag_0.7_ + Au_0.7_Ag_0.3_, determined using EDS.

## Appendix A. Modeling Details

For nanoparticles in this study, classical electromagnetic theory can adequately model the light–nanoparticle interaction, as quantum effects are negligible. The nanoparticle systems were modeled on a FEM-based framework with COMSOL Multiphysics, which numerically solves the frequency domain form of the Maxwell’s equations:

(A1) $$\nabla \times (\mu_{r}{}^{-1}\nabla \times E_{sc})-k_{0}{}^{2}(\varepsilon_{r}-j\frac{\sigma }{\omega \varepsilon_{0}})E_{sc}=0$$

with *k*_0_ the wave number, *E_sc_* the scattered field, and *μ_r_*, *ε_r_*, and *σ* representing the properties of the material, namely, relative permeability, relative permittivity, and electrical conductivity, respectively.

Equation (A1) was solved for the scattered field, *E_sc_*, in the presence of nanoparticles for a plane-polarized background electric field, *E*_0_, in spherical computational domains (radius: 800 nm) surrounded by a perfectly matched layer (thickness: 200 nm) that completely absorbed any radiation. Thus, the total field *E* was the superposition of the scattered and incident electric fields, *E_sc_* and *E*_0_, respectively. The perfectly matched layer was discretized by prismatic elements of five layers, while the rest of the computational domain with the nanoparticles was discretized by tetrahedral elements. A grid independence study was carried out by varying the number of grid elements and comparing local electric field to ensure that the mesh elements are small enough for numerical accuracy.
