# Supplementary material for: Layer-by-Layer-Stabilized Plasmonic Gold-Silver Nanoparticles on TiO2: Towards Stable Solar Active Photocatalysts
Source: Nanomaterials (Basel). 2021 Oct 6;11(10):2624. doi: 10.3390/nano11102624 (PMC8540643; doi:10.3390/nano11102624)
Supplement: Supplementary file 1 [file nanomaterials-11-02624-s001.zip › nanomaterials-1382109-supplementary.pdf]

Supplementary Materials

Layer-by-Layer-Stabilized Plasmonic Gold-Silver Nanoparticles on TiO_2: Towards Stable Solar Active Photocatalysts

Fons Dingenen [1,2], Natan Blommaerts [1,2], Myrthe Van Hal [1,2], Rituraj Borah [1,2], Daniel Arenas-Esteban [2,3], Sara Bals [2,3], Silvia Lenaerts [1,2] and Sammy W. Verbruggen [1,2,*]

[1] Sustainable Energy, Air & Water Technology (DuEL), University of Antwerp, Groenenborgerlaan 171, 2020 Antwerp, Belgium; fons.dingenen@uantwerpen.be (F.D.); natan.blommaerts@uantwerpen.be (N.B.); myrthe.vanhal@uantwerpen.be (M.V.H.); rituraj.borah@uantwerpen.be (R.B.); silvia.lenaerts@uantwerpen (S.L.); sammy.verbruggen@uantwerpen.be (S.W.V.)

[2] NANOlab Center of Excellence, University of Antwerp, Groenenborgerlaan 171, 2020 Antwerp, Belgium

[3] Electron Microscopy for Materials Science, University of Antwerp, Groenenborgerlaan 171, 2020 Antwerp, Belgium; daniel.arenasesteban@uantwerpen.be (D.A.E.); sara.bals@uantwerpen.be (S.B.)

* Correspondence: Correspondence: sammy.verbruggen@uantwerpen.be

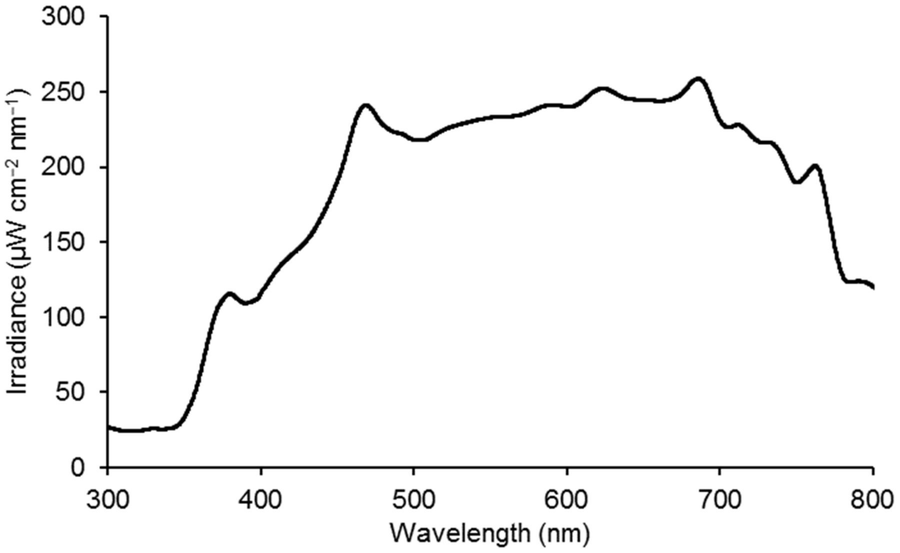

Figure S1. The irradiance spectrum of the used solar simulator.

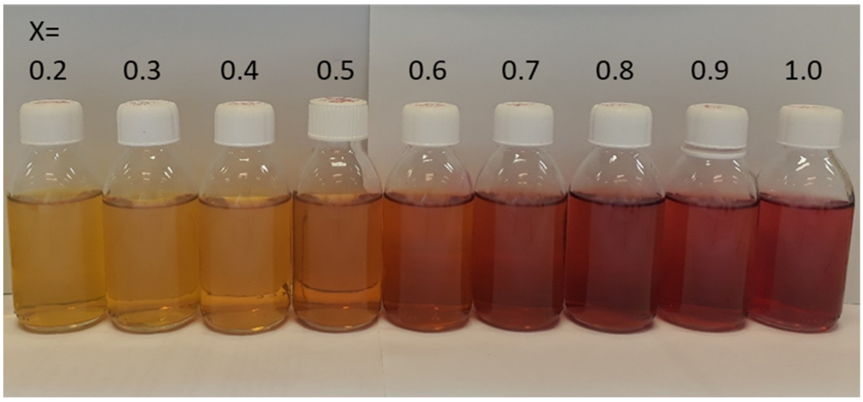

Figure S2. The different Au_xAg_{1-x} NP suspensions.

(a)

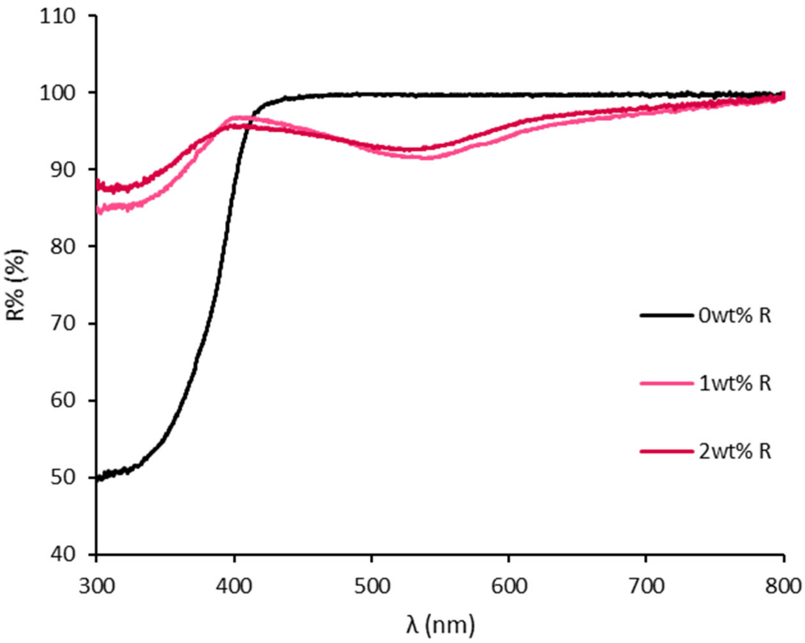

110
100
90
80
70
60
50
40
R% (%)
300
400
500
600
700
800
λ (nm)
0wt% R
1wt% R
2wt% R

(b)

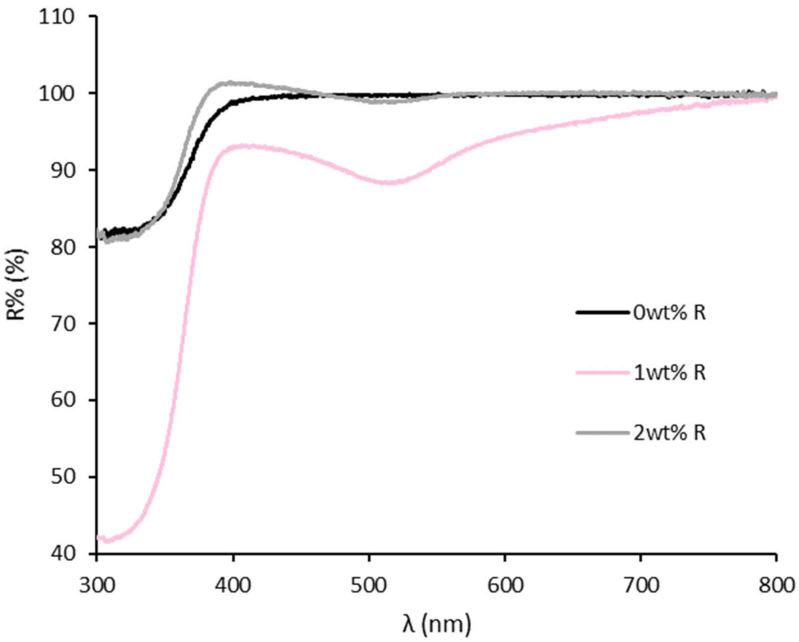

110
100
90
80
70
60
50
40
R% (%)
300
400
500
600
700
800
λ (nm)
0wt% R
1wt% R
2wt% R

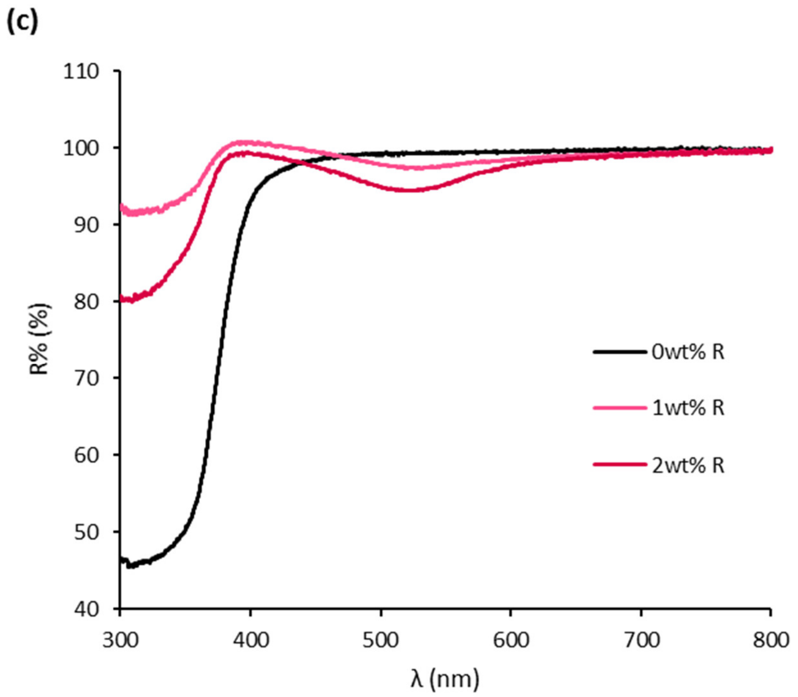

Figure S3. DRS spectra for (**a**) pure anatase species, (**b**) PC500 species and (**c**) P25 species, plotting the fraction of reflectance (R%) against the wavelength of the incident light (λ). The colors of the plasmon modified species are realistic, with exception of 0 wt.% R (black). Note that only the relative differences should be considered, since the absolute values depend on the added amount of photocatalyst. Hence, the VIS absorption of PC500 + 1 wt.% R is still rather small due to the failed photo-impregnation.

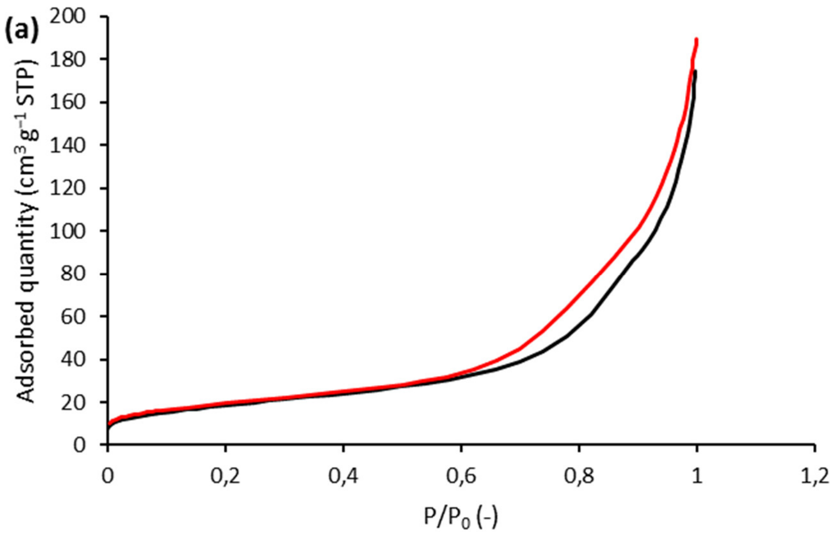

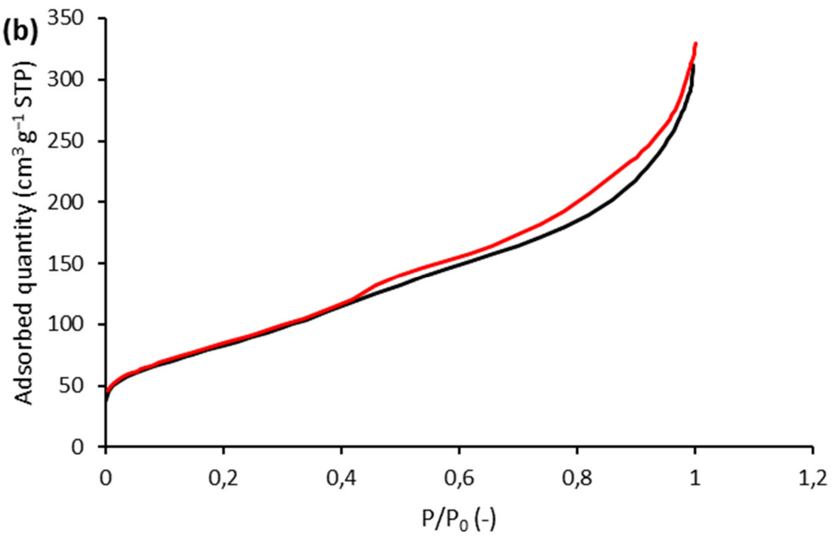

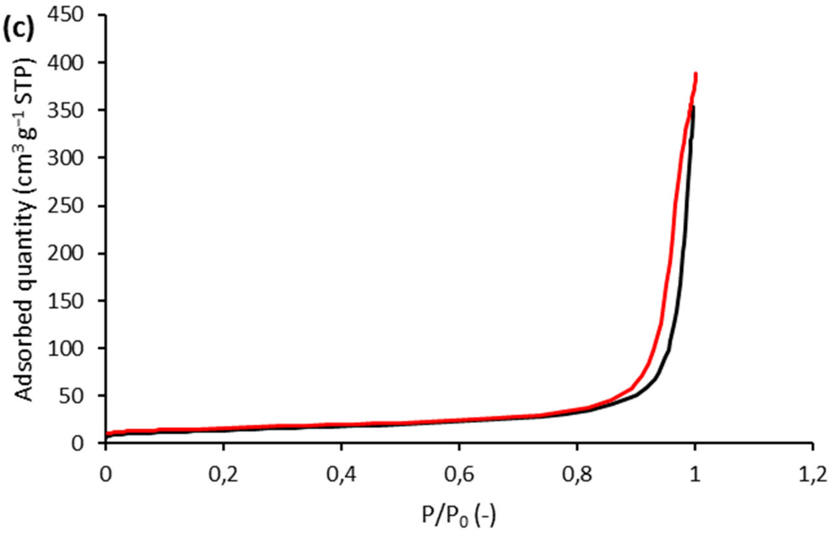

Figure S4. N_2 ad- (black) and desorption (red) curve, plotting the adsorbed quantity (in cm^3.g at standard temperature and pressure (STP)) vs. the pressure ratio (P/P_0), for a representative (**a**) pure anatase, (**b**) PC500 and (**c**) P25 sample. All the other plasmon modified samples gave similar curves. For sake of simplicity, only these 3 are given.

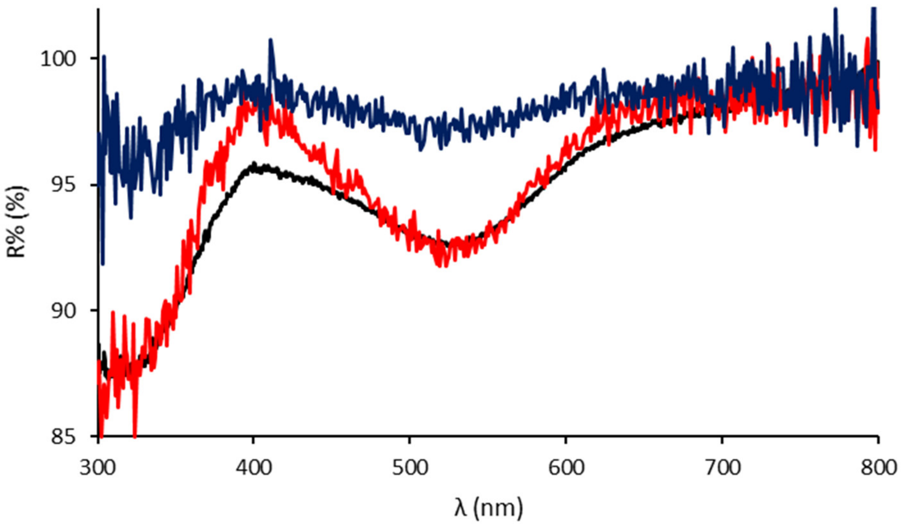

Figure S5. DRS spectra for P25 + 2 wt.% R (black), + 3 wt.%. R (red) and + 5 wt.% R (blue), plotting the fraction of reflectance (R%) against the wavelength of the incident light (λ). The rather high R% values are due to the small amounts used.

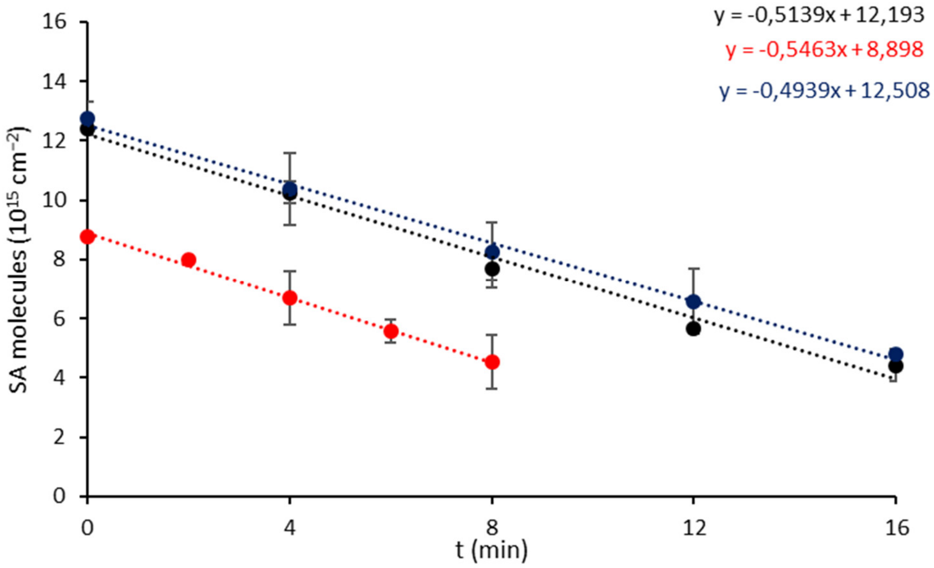

Figure S6. SA degradation curves, plotting the number of SA molecules per cm^2 against the time (t) for P25 + 2 wt.% R (black), + 3 wt.% R (red) and + 5 wt.% R (blue).

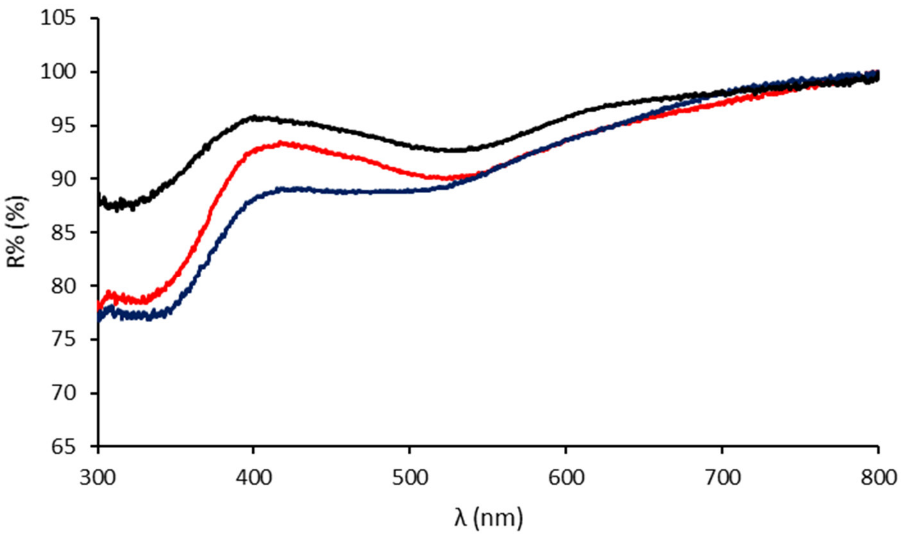

Figure S7. DRS spectra for P25 + 2 wt.% R (black) and P25 + 2 wt.%. non- (red) and LbL stabilized (blue) $Au_{0.3}Ag_{0.7}$ + $Au_{0.7}Ag_{0.3}$, plotting the fraction of reflectance (R%) against the wavelength of the incident light (λ). The rather high R% values are due to the small amounts used.

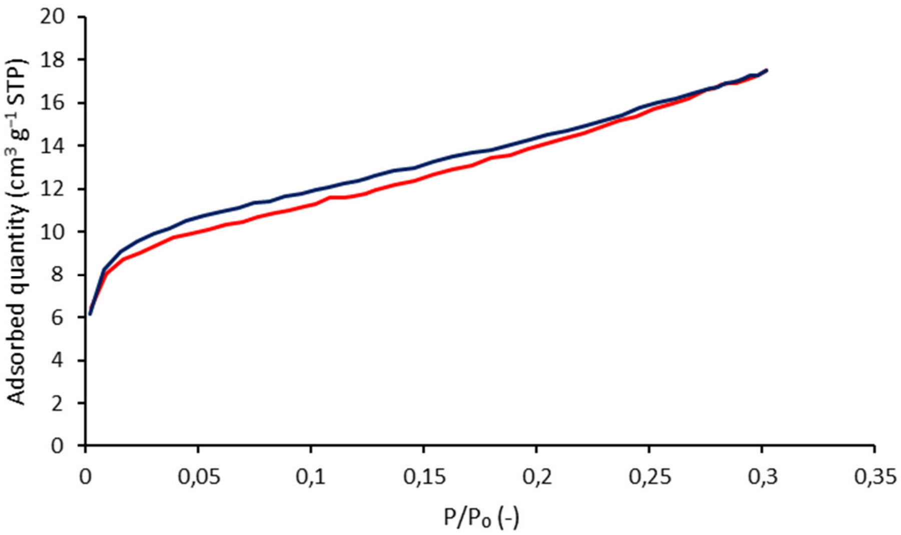

Figure S8. N_2 adsorption curve for both P25 + 2 wt.% non- (red) and LbL stabilized (blue) $Au_{0.3}Ag_{0.7}$ + $Au_{0.7}Ag_{0.3}$, plotting the adsorbed volume (V) at standard temperature and pressure (P/P_0) (room temperature and 1 atm) against the relative pressure (ranging between 0 and 0.3).

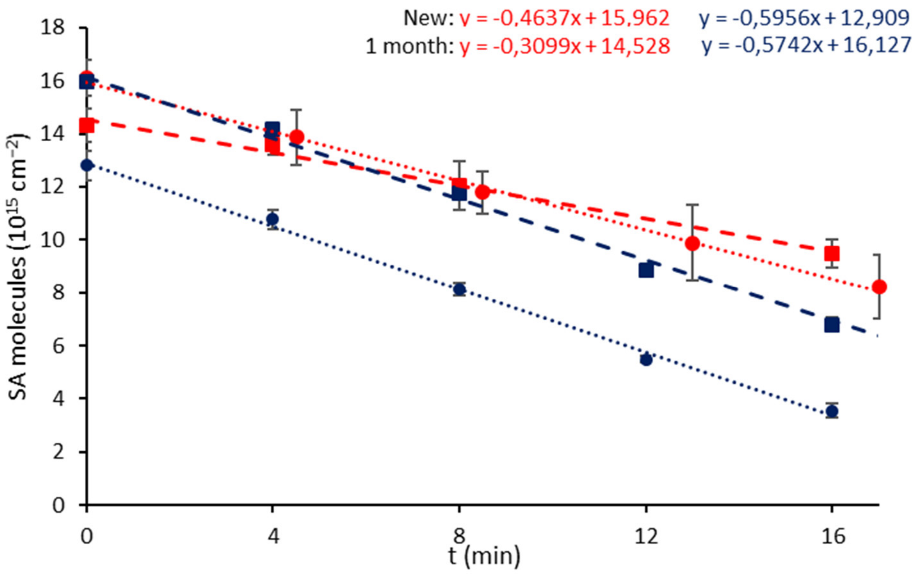

Figure S9. SA degradation curves, plotting the number of SA molecules per cm^2 against the time (t) for P25 + 2 wt.% R (black) and a representative P25 + 2 wt.% $Au_{0.3}Ag_{0.7}$ + $Au_{0.7}Ag_{0.3}$ sample (red) and the LbL stabilized equivalent (blue) samples. The latter two are tested both new (circles, dotted line) and after 1 month in darkness (squares, dashed line).

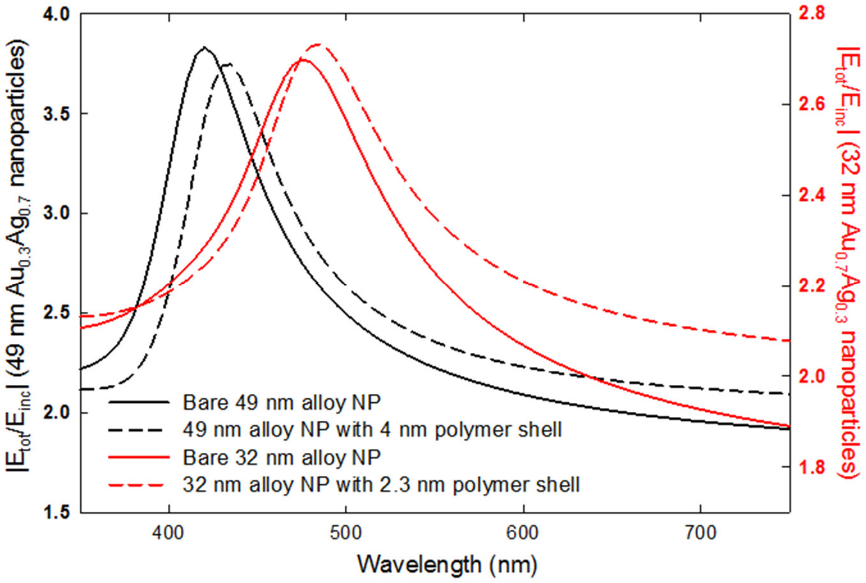

Figure S10. Near field enhancement (NFE), expressed as the ratio between the generated electrical near field and the incoming electrical field ($|E_{tot}/E_{inc}|$), for both isolated original (full) and LbL-stabilized (dashed) $Au_{0.3}Ag_{0.7}$ (black) and $Au_{0.7}Ag_{0.3}$ (red), plotted against the wavelength of the incoming light. The NFE is averaged over the NP surface. Due to the fact that the NPs are modelled as perfect alloys, the spectra are slightly blue-shifted compared to the experimental absorbance spectra, even though a small red shift would be expected for NFE spectra. Note furthermore that these maximal values do not necessarily correspond to the most efficient wavelengths to enhance P25's photocatalytic activity. This activity originates both from UV and VIS light due to P25's gap states.

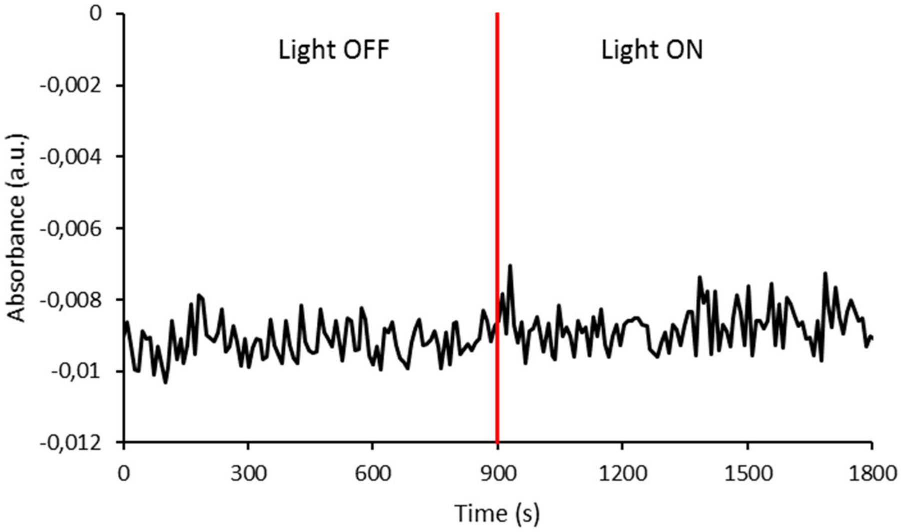

Figure S11. CO_2 FTIR absorbance plotted against time in a closed flat bed reactor with P25 + 2 wt.% LbL-$Au_{0.3}Ag_{0.3}$ + -$Au_{0.7}Ag_{0.3}$ (44 µg cm^{-2}, 3 cm x 1.5 cm) for a 497 mL min^{-1} air flow. After 15 min (red line), the reactor was illuminated with 1 sun AM1.5G simulated solar light. Note that the generated amount of CO_2 was far less than 1 ppm min cm^{-2}.

Table S1. Theoretical gold fraction ($Au\%_{th}$) and experimental gold ($Au\%_m$) and silver fractions ($Ag\%_m$) for the different Au_xAg_{1-x} NP suspensions.

$Au\%_{th}$ (%)	$Au\%_m$ (%)	$Ag\%_m$ (%)
20	18	82
30	31	69
40	40	60
50	52	48
60	61	39
70	69	31
80	80	20
90	90	10
100	100	0

Table S2. Used metal masses, corresponding costs for a Au and Ag precursor price of resp. €91.6 g^{-1} and €2.68 g^{-1}, TOFs and ICERs for the different metal loadings of P25 (for 1 m^2 with a photocatalyst loading of 44 µg cm^{-2}).

Metal loading (wt %)	m_{Au} (mg)	m_{Ag} (mg)	p_{AuAg} (euro)	TOF (10^{18} cm^{-2} h^{-1})	ICER (10^{-21} euro h)
0	0	0	0	19.97	X
1	3.22	1.18	0.60	24.83	12.27
2	6.45	2.35	1.19	31.15	10.67
3	9.67	3.53	1.79	33.21	13.51
5	16.12	5.88	2.98	29.94	29.92

Table S3. Experimental (Exp.) atomic fraction and experimental and theoretical (Th.) mass fraction for O, Ti, Ag and Au in P25 + 2 wt.% LbL $Au_{0.3}Ag_{0.7}$ + $Au_{0.7}Ag_{0.3}$, determined using EDS.

Element	Exp. atomic fraction (mol %)	Exp. mass fraction (wt %)	Th. Mass fraction (wt %)
O	54 ± 4	28 ± 3	39
Ti	46 ± 4	70 ± 3	58
Ag	0.14 ±0.03	0.049 ± 0.08	0.7
Au	0.17 ± 0.2	1.06 ± 0.07	1.3
